# Electrophoretic profiles of lipopolysaccharides from *Rhizobium* strains nodulating *Pisum sativum* do not reflect phylogenetic relationships between these strains

**DOI:** 10.1007/s00203-017-1374-1

**Published:** 2017-04-06

**Authors:** Jolanta Kutkowska, Monika Marek-Kozaczuk, Jerzy Wielbo, Marek Wójcik, Teresa Urbanik-Sypniewska

**Affiliations:** Department of Genetics and Microbiology, Maria Curie-Skłodowska University, Lublin, Poland

**Keywords:** *Rhizobium leguminosarum*, *Pisum sativum*, AFLP fingerprinting, 16S rRNA, Lipopolysaccharides, Fatty acids

## Abstract

Rhizobia that nodulate peas comprise a heterogeneous group of bacteria. The aim of this study was to investigate the relationship between phylogeny and electrophoretic and hydroxy fatty acid lipopolysaccharide (LPS) profiles of pea microsymbionts. Based on amplified fragment length polymorphism (AFLP) fingerprinting data, the pea microsymbionts were grouped into two clusters distinguished at 58% similarity level. Based on the concatenated 16S rRNA, *recA*, and *atpD* housekeeping gene data, the microsymbionts appeared to be most closely related to *Rhizobium leguminosarum* biovars *viciae* and *trifolii*. Applying cluster analysis to their LPS electrophoretic profiles, the strains were assigned to two major groups with different banding patterns. All hydroxy fatty acids common to *R. leguminosarum* and *R. etli* were detected in each examined strain. Differences in the proportions of 3- to ω-1 hydroxy fatty acids allowed us to distinguish two groups of strains. This classification did not overlap with one based on LPS electrophoretic profiles. No clear correlation was apparent between the genetic traits and LPS profiles of the pea nodule isolates.

**Electronic supplementary material:**

The online version of this article (doi:10.1007/s00203-017-1374-1) contains supplementary material, which is available to authorized users.

## Introduction

Biological nitrogen fixation is one of the most important processes whereby reduced nitrogen compounds are introduced into the biosphere. Bacteria from the order Rhizobiales, both free-living and those forming a symbiotic relationship with legume plants, are capable of fixing atmospheric nitrogen (Zahran 1999). One of the striking features of symbiosis is the relatively high specificity of the interaction between the microsymbiont and the host plant. Most rhizobia have a narrow host range and form nodules on well-defined plant species, e.g., *Rhizobium leguminosarum* bv. *viciae* is a symbiont of the legumes of the tribe Vicieae, which includes the genera *Vicia*, *Pisum*, *Lathyrus*, and *Lens,* while the symbiosis of *Rhizobium* bv. *trifolii* is confined to *Trifolium* plants (Tian et al. 2010; Kumar et al. 2015).

Nodule development requires the exchange of molecular signals between the two partners, flavonoids (produced by plants) and Nod factors (produced by bacteria) that are recognized by plant receptors. This leads to the expression of plant genes, cell de-differentiation, organogenesis, and infection of root nodules (Skorupska et al. 2006; Zgadzaj et al. 2015).

In addition, partner recognition and effective symbiosis require an appropriate structure of surface polysaccharides, such as lipopolysaccharides (LPS), exopolysaccharides (EPS), external capsular polysaccharides (CPS or K-antigen polysaccharides, KPS), as well as periplasmic cyclic β-glucans, high molecular weight neutral polysaccharide (glucomannan), and gel-forming polysaccharide (GPS) (Laus et al. 2006; Janczarek 2011; Kawaharada et al. 2015).

The importance of the different types of polysaccharides in the nodulation process varies depending on the type of nodules (determinate or indeterminate). For instance, acidic EPS secreted into the extracellular environment is especially significant in the establishment of effective symbiosis with host plants that form indeterminate nodules (Hotter and Scott 1991). On the other hand, the presence of the O-chain portion of LPS is required for effective symbiosis in both determinate and indeterminate (Priefer 1989) nodule-forming hosts (Noel et al. 1986).

Metabolism-related traits and physiological characteristics are quite often used to study the diversity of rhizobia (Dresler-Nurmi et al. 2009). Native rhizobial populations are diverse and contain strains differing in their physiological features, genomic structure, and the efficiency of nitrogen fixation (Wielbo et al. 2010, 2011; Kumar et al. 2015). Accordingly, some LPS traits, such as their electrophoretic profile and fatty acid composition, may be used for taxonomic classification (Santamaria et al. 1997). The composition of both total cellular fatty acids and LPS fatty acids has been used for bacterial identification and taxonomy (Yokota and Sakane 1991; Dresler-Nurmi et al. 2009; Choma and Komaniecka 2011).

Recent studies have revealed particularly high diversity in the genome organization and metabolic versatility of *R. leguminosarum* isolates. Kumar et al. (2015) demonstrated that the diversity of *R. leguminosarum* within a local population of nodule isolates was 10 times higher than that found in *Ensifer medicae*.

The molecular methods used for the classification of bacterial species include 16S rRNA gene sequencing and phylogenetic analysis of housekeeping genes (Ramírez-Bahena et al. 2008). Amplified fragment length polymorphism (AFLP) analysis has also been used to evaluate the genomic diversity of nodule bacteria (Wolde-Meskel et al. 2004; Wdowiak-Wróbel and Małek 2005).

The aim of the current study was to investigate the diversity of strains nodulating *Pisum sativum* according to genetic markers and phenotypic properties. 16S rRNA, *recA*, and *atpD* gene sequences were used as genetic markers, and were also profiled by AFLP. The phenotypical assessment was based on electrophoretic patterns and fatty acid composition of LPS, and on EPS production. Additionally, such physiological criteria as sensitivity to salt, detergents, pH, and elevated temperature were studied.

## Materials and methods

### Bacterial strains and growth conditions

All strains used in this study (15 strains) were obtained from surface-sterilized nodules of pea (*P. sativum*) grown in arable soil in the region of Lublin, Poland, as described earlier (Wielbo et al. 2010, 2011). The isolated strains were reinoculated onto *P. sativum* cv. Ramrod to confirm their nodulation ability in sterile conditions. For further studies, the bacteria were grown either on yeast mannitol agar (79CA) plates (Vincent 1970) or in liquid 79CA medium, with 1% glycerol as a carbon source, at 28 °C with shaking (160 rpm).

### AFLP analyses

The genome diversity of rhizobial strains was studied using a modified AFLP method, as described by Wdowiak-Wróbel and Małek (2005). Briefly, genomic DNA was digested with *Pst*I and ligated to adaptors *Pst*_AR (5′-TGTACGCAGTCTACG-3′) and *Pst*_AF (5′-CTCGTAGACTGCGTACATGCA-3′) for 4 h at 37 °C. Next, it was subjected to a selective PCR using a *Pst*I-GCG primer containing additional GCG nucleotides at the 3′-terminus (5′-GACTGCGTACATGCAGGCG-3′). The amplified products were separated by electrophoresis in 2% (w/v) agarose gel in Tris–borate-EDTA (TBE) buffer. AFLP profiles were used for the construction of a dendrogram, employing the unweighted pair-group method with arithmetic mean (UPGMA) clustering method in STATISTICA v. 10 for Windows (StatSoft Inc., Tulsa, OK).

### PCR amplification and gene sequencing

Genomic DNA was extracted and purified as described previously (Marek-Kozaczuk et al. 2013). The 16S rRNA was amplified using 16S63f (5′-CAGGCCTAACACATGCAAGTC-3′) and 16S1387r (5′-GGGCGGWGTGTACAAGGC-3′) primers (Marchesi et al. 1998). The primers and protocols used for the amplification and sequencing of chromosomal housekeeping genes *atpD*, encoding the beta subunit of ATP-synthase (Bailly et al. 2007), and *recA*, encoding a DNA recombinase (Gaunt et al. 2001), were described earlier (Marek-Kozaczuk et al. 2013).

Sequencing was performed using the BigDye^®^ Terminator Cycle Sequencing Kit (Applied Biosystems, Carlsbad, CA, USA) and an ABI Prism 3730 XL Genetic Analyzer (Applied Biosystems). The sequences were aligned with those deposited in the GenBank using the MEGA5.05 software package (Tamura et al. 2011). Distances were calculated according to Kimura’s two-parameter model of substitutions (Kimura 1980). Phylogenetic trees were constructed using the neighbor-joining (NJ) method. Bootstrap analysis was based on 1000 replications (Felsenstein 1985).

### Nucleotide sequence accession numbers

The sequences have been submitted to the GenBank database under accession numbers KJ528929–KJ528943 for 16S rRNA genes; KJ528903–KJ528911, KJ493937–KJ493941, and KJ481208 for *atpD*; and KJ528913–KJ528927 for *recA* genes.

### Sodium dodecyl sulfate (SDS)-tricine-polyacrylamide gel electrophoresis (PAGE)

LPS was prepared by whole-cell microextraction using proteinase K digestion. Bacterial cells were washed three times with 0.5 M NaCl, and wet cell mass samples (50 mg) were solubilized in 200 μL of lysing buffer according to Apicella (2008). Samples (5 μL) were loaded onto 12.5% SDS-PAGE gel. Electrophoresis was carried out using a tricine buffer system. LPS profiles were visualized as described by Tsai and Frasch (1982). The UPGMA method was used to construct a dendrogram of LPS profiles. The presence of a band was scored as 1 and its absence as 0. Bands with the same mobility, regardless of their intensity, were considered to be identical.

### Analysis of LPS-derived fatty acids

Fatty acids were released (4 M HCl, 100  °C, 4.5 h) from LPS samples obtained from whole cell lysates. Free fatty acids were esterified by methanolysis (1 M methanolic HCl, 80 °C, 1.5 h). The samples were trimethylsilylated (TMS) with a Sylon HTP kit (Supelco, Bellefonte, PA, USA) for 30 min at room temperature. Methyl esters of nonpolar fatty acids and TMS ethers of hydroxy fatty acids were identified by gas chromatography–mass spectrometry (GC–MS) on an Agilent gas chromatograph (7890A, Santa Clara, CA, USA) equipped with a capillary column (HP-5MS, 30 m × 0.25 mm, Supelco, Bellefonte, PA, USA) connected to a mass selective detector (MSD5975, Agilent Technologies, Santa Clara, CA, USA).

Fatty acid weight percentage of total methyl esters was analyzed with the UPGMA method using unweighted pair-group average Euclidean distances, in the STATISTICA package.

### Quantitation of EPS production

Three-day 79CA medium cultures of rhizobia were centrifuged at 14,000×*g* for 30 min, and EPS was precipitated from supernatants with three volumes of cold ethanol. The quantification of total carbohydrates, expressed as glucose equivalents, was performed by the anthrone method (Yasar 2005). The EPS yield was expressed as mg of EPS produced per mg of bacterial protein. Total protein was determined by the Lowry method after overnight solubilization of bacteria in 0.1 M NaOH and 5.0% SDS at room temperature.

### Sensitivity to salt, detergents, pH, and elevated temperature

The ability of strains to grow in the presence of saline was determined in liquid 79CA medium supplemented with 0.01, 0.1, 1.0, and 3.0% (w/v) of NaCl. The bacterial growth was measured as optical density at 550 nm (OD_550_). The growth of each strain was tested at pH 4.5, 5, 7.2, 7.5, and 8.0, in liquid 79CA medium. Temperature ranges and growth optima of the isolates were determined by incubating the inoculated 79CA liquid medium cultures at 20, 28, 37, and 42 °C. Cell viability was tested by spreading 100 µL of undiluted cultures on 79CA agar plates.

Sensitivity to SDS and sodium deoxycholate (DOC) was determined using a dilution method in 96-well microtiter plates with serially diluted detergents at concentrations from 0.001 to 0.25% (w/v) in 79CA medium. The minimum inhibitory concentration (MIC) was defined as the lowest concentration of the detergents at which no bacterial growth was visible.

All samples were incubated for 3 days. The experiments were performed in triplicate.

Phenotypic data (sensitivity to salt, SDS, DOC, and pH) were analyzed by the UPGMA method using weighted pair-group average Euclidean distances.

## Results

### Analysis of the genetic diversity of pea nodule isolates

**Fig. 1:**
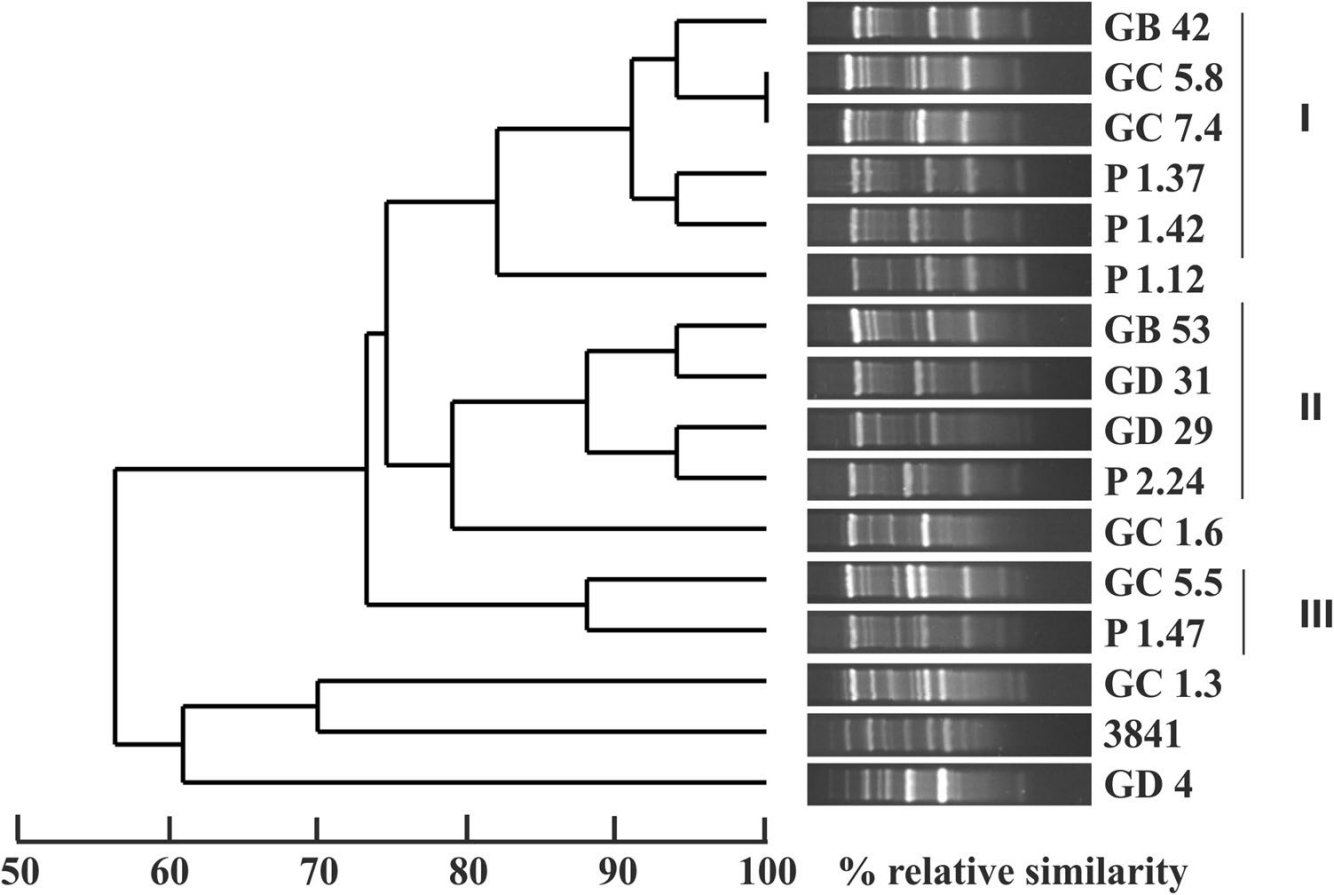
Genetic diversity of pea and field pea rhizobia isolated from one region of Poland, as revealed by AFLP fingerprinting. The dendrogram was constructed using the UPGMA method. AFLP clusters defined at 85% similarity levels are indicated by numbers (*I*–*III*)

Based on cluster analysis of AFLP fingerprints with the unweighted pair-group method, all strains were grouped into two clusters at 58% similarity level (Fig. 1). Only two strains, GC 5.8 and GC 7.4, had identical DNA fingerprints. Strains GC 1.3 and GD 4 shared the highest similarity with the reference strain *R. leguminosarum* bv. *viciae* 3841 with 70 and 61% similarity, respectively. Strain GD 4 was most distant from all isolates. At the cut off value of 85%, the strains could be subdivided into three subgroups: group I—strains GB 42, GC 5.8, GC 7.4, P 1.37, and P 1.42, displaying 92% similarity; group II—strains GB 53, GD 31, GD 29, and P 2.24; and group III—formed by strains GC 5.5 and P 1.47, with 88% similarity.

The 16S rRNA gene-based phylogenetic tree was less discriminative and showed that the analyzed 15 pea microsymbionts clustered in a well-resolved branch with *R. leguminosarum* bv. *viciae* strains USDA 237 and 3841, and *R. leguminosarum* bv. *trifolii* ATCC 14480 with bootstrap support of 98% (Supplemental Fig. 1).

**Fig. 2:**
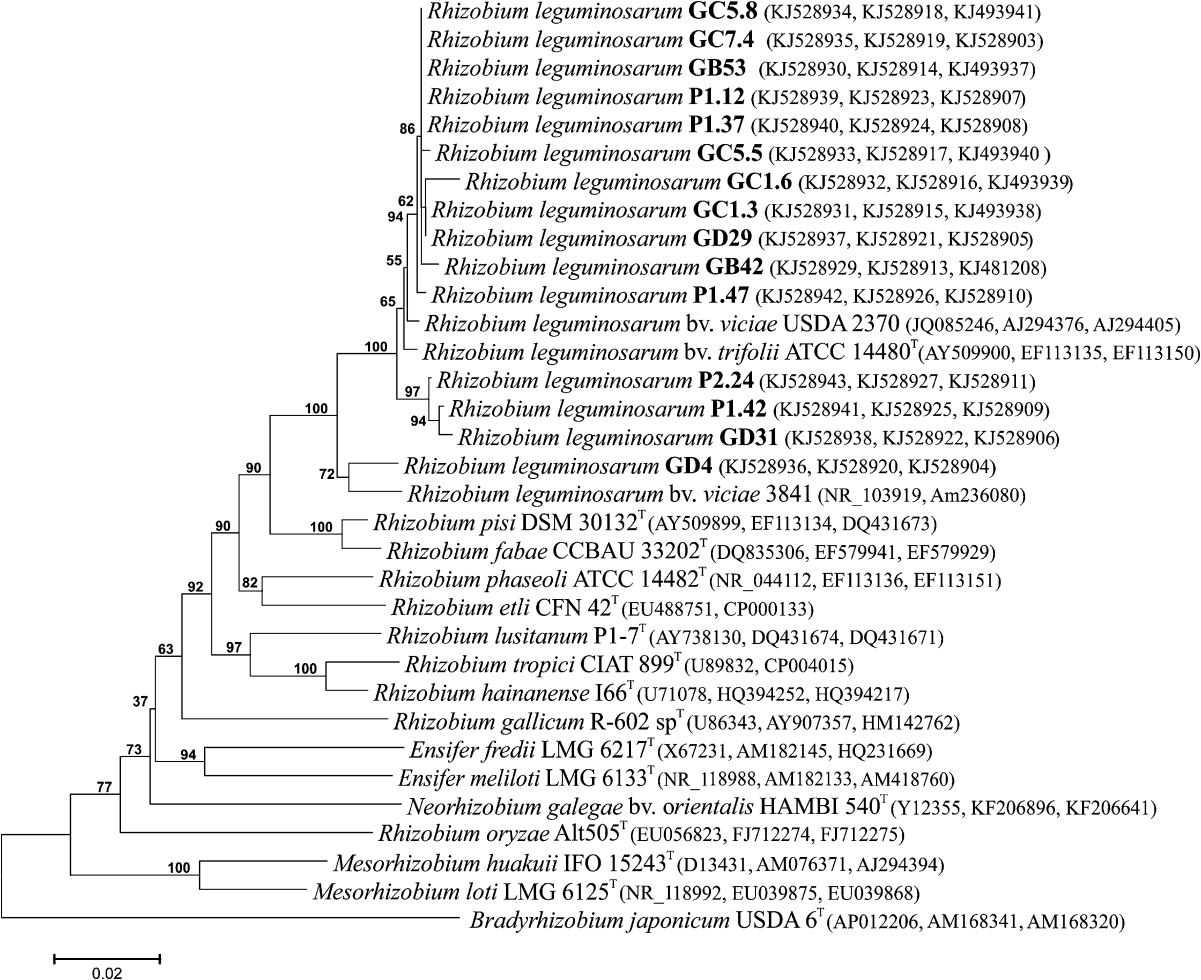
Neighbor-joining phylogenetic tree based on combined partial 16S rRNA, *recA*, and *atpD* sequences of strains from pea nodules and of closely related *Rhizobium* species. Bootstrap analysis was based on 1000 resamplings. Bar, number of nucleotide substitution per site

To further classify the isolates, the *recA* and *atpD* core genes were sequenced. The similarity values for the *recA* and *atpD* genes were high and ranged from 95.1 to 100% and from 92.7 to 100%, respectively (Supplemental Figs. 2, 3). The phylogenetic tree constructed on the basis of the concatenated genes 16S rRNA, *recA*, and *atpD* showed that all strains except GD 4 formed a cluster with the reference strains *R. leguminosarum* bv. *trifolii* ATCC 14480 and *R. leguminosarum* bv. *viciae* USDA 237 (Fig. 2) and confirmed that the *P. sativum* isolates were phylogenetically related to the biovars *viciae* and *trifolii* of *R. leguminosarum*. Isolate GD 4 showed the highest similarity, i.e., 98% sequence identity of combined gene sequences to *R. leguminosarum* bv. *viciae* 3841. A distinct branch at 99.5–99.6% sequence identity of the concatenated genes was formed by the three microsymbionts GD31, P1.42, and P2.24.

### Electrophoretic analysis of LPS

**Fig. 3:**
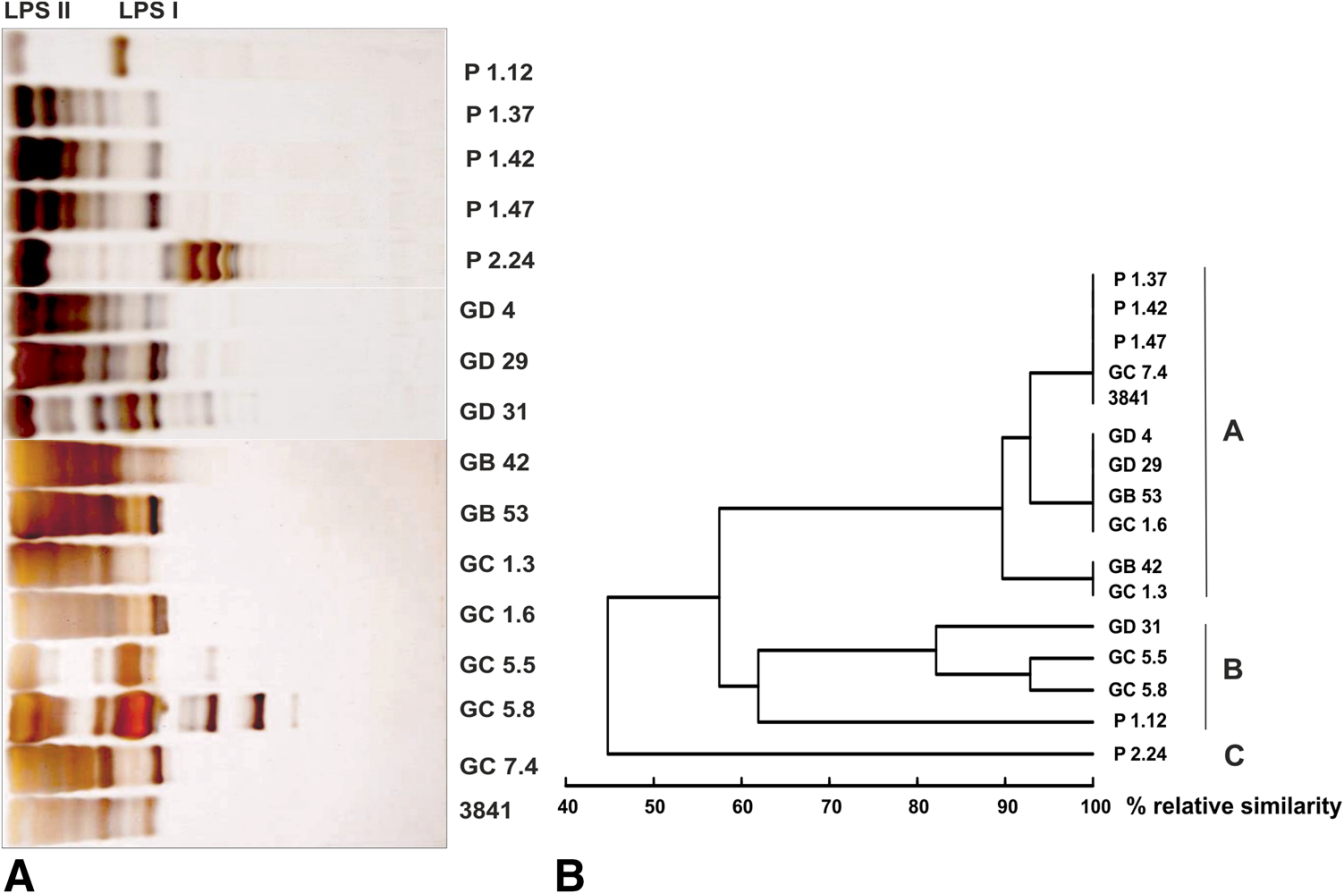
**a** Tricine SDS-PAGE profiles of LPS of strains isolated from pea plant nodules and the reference strain *R. leguminosarum* bv. *viciae* 3841. **b** Dendrogram constructed using data from **a**

Based on LPS electrophoretic profiles, the strains isolated from pea nodules were grouped into two major clusters at a similarity level of 58%, as determined by the UPGMA method (Fig. 3a, b). The largest cluster (cluster A) contained 10 isolates, including the reference strain 3841 (Table 1), with the LPS profile consisting of two major bands representing high molecular weight LPS I, i.e., LPS containing the O-polysaccharide, and LPS II, a low molecular weight LPS that contains lipid A and a core oligosaccharide (D’Heaze et al. 2007) (Fig. 3a). Four isolates, P 1.37, P 1.42, P 1.47, and GC 7.4, had the same LPS profile as strain 3841. Strains GB 42 and GC 1.3 formed a subcluster with 90% similarity, characterized by a less prominent LPS I region.

**Table 1:** NaCl and pH tolerance, sensitivity to detergents, electrophoretic LPS pattern, and EPS production of *Pisum sativum* isolates compared with reference strain 3841

LPS pattern	Strain	Range of		MIC % (w/v)		EPS (µg mg^−1^)
		NaCl (%)	pH	DOC	SDS	
A	P 1.37	0.01–0.1	4.5–7.5	0.12 ± 0.015	0.06 ± 0.005	287.5 ± 20.4
	P 1.42	0.01–1.0	4.5–7.5	0.12 ± 0.02	0.015± 0.002	453 ± 40.2
	P 1.47	0.01–1.0	4.5–7.5	0.06 ± 0.002	0.12 ± 0.03	330.4 ± 25.1
	GC 7.4	0.01–1.0	4.5–8.0	0.12 ± 0.015	0.06 ± 0.006	240.5±26.4
	3841	0.01–1.0	4.5–7.5	0.06 ± 0.005	0.06 ± 0.005	136.4 ± 18
	GD 4	0.01–1.0	4.5–8.0	0.12 ± 0.02	0.03 ± 0.002	270 ± 21
	GD 29	0.01–3.0	4.5–8.0	0.12 ± 0.025	0.015 ± 0.002	311.3 ± 16.3
	GB 53	0.01–3.0	4.5–8.0	0.12 ± 0.035	0.015 ± 0.003	235.6 ± 21.3
	GC 1.6	0.01–3.0	4.5–7.5	0.12 ± 0.03	0.12 ± 0.02	216.7 ± 15.1
	GB 42	0.01–1.0	4.5–7.5	0.12 ± 0.03	0.03 ± 0.003	347.4 ± 45
	GC 1.3	0.01–3.0	4.5–8.	0.06 ± 0.004	0.015 ± 0.002	298.6 ± 33.1
B	GD 31	0.01–1.0	4.5–8.0	0.25 ± 0.025	0.06 ± 0.005	401.7 ± 15.16
	GC 5.5	0.01–1.0	7.2–8.0	0.015± 0.002	0.03 ± 0.004	113.7 ± 16.3
	GC 5.8	0.01–1.0	4.5–8.0	0.25 ± 0.03	0.06 ± 0.004	300.6 ± 22
	P 1.12	0.1–1.0	4.5–7.5	0.06 ± 0.006	0.06 ± 0.005	332.7 ± 18.2
C	P 2.24	0.01–1.0	4.5–7.0	0.06 ± 0.005	0.06 ± 0.004	260 ± 26.4

NaCl concentrations and pH range of culture medium for which bacterial growth was detected; MIC, minimum inhibitory concentration; DOC, sodium deoxycholate; SDS, sodium dodecyl sulfate. Exopolysaccharide (EPS) production is expressed as µg of glucose (Glc) equivalents mg^−1^ protein. The data presented are averages (± standard deviation) of three independent measurements

In the other cluster (cluster B), LPS of strains GC 5.5, GC 5.8, and GD 31 (Table 1) was characterized by a much higher heterogeneity of slow- and fast-migrating bands, whereas the LPS of strain P 1.12 migrated as two major bands. The LPS of strain P 2.24 had a distinct pattern and was categorized as type C (Table 1).

Four isolates (P1.37, P1.42, P1.47, and GC7.4) with LPS patterns similar to that of 3841 (Fig. 3) were more closely related to strains *R. leguminosarum* bv. *viciae* USDA 2370 and *R. leguminosarum* bv. *trifolii* ATCC 14480 than to strain 3841, while isolate GD 4, with the highest similarity to strain 3841, had a different LPS profile.

### Fatty acid analysis

Strong acidic hydrolysis of LPS preparations was used to release both ester- and amide-bound fatty acids. The analysis revealed the presence of the following hydroxy fatty acids in all strains isolated from pea nodules and *R. leguminosarum* bv. *viciae* 3841: 3-hydroxymyristic (C_14:0_ 3-OH), 3-hydroxypentadecanoic (C_15:0_ 3-OH) 3-hydroxypalmitic (C_16:0_ 3-OH), 3-hydroxystearic (C_18:0_ 3-OH), and (*ω*-1) very long chain 27-hydroxyoctacosanoic (C_28:0_ 27-OH) acids, which are specific to *R. leguminosarum* (Vedam et al. 2003; Brown et al. 2011) and *R. etli* lipid A (Que et al. 2000) (Table 2). In addition, 29-hydroxytriacontanoic acid (C_30:0_ 29-OH) was detected. The presence of this fatty acid in lipid A of strain 3841 was also reported by Vedam et al. (2003). The fatty acid ratio varied among the strains; different ratios of primary acyl chains (ester- and amide-linked to the lipid A backbone) and secondary fatty acids (substituting hydroxyl groups of the primary acyl chains) were found. Based on fatty acid composition, two discrete profile groups could be distinguished (Table 2).

**Table 2:** Composition of LPS-derived fatty acid from pea nodule isolates and strain 3841

Strain	% Fatty acids					
	C_14:0_ 3-OH	C_15:0_ 3-OH	C_16:0_ 3-OH	C_18:0_ 3-OH	C_28:0_ 27-OH	C_30:0_ 29-OH
P 1.47*	20	4.8	13.2	20.5	36	5.5
GD 4*	33	6	8	29	22	2
GC 1.3*	25.2	5.3	9	32.1	26.3	1.8
GC 5.8*	15.5	5	4	28	35	2.5
3841*	35	5	9	19	30	2
P 1.12	37	3	30.4	23.6	5	1
P 1.37	32.7	5.2	22.7	35.3	3.1	1
P 1.42	38.8	4	30	22.2	4.5	0.5
P 2.24	30	3.7	30.5	32	3	0.8
GD 29	32.7	4.5	34.5	24.3	3.5	0.5
GD 31	36	3.5	38.1	18.4	3	1
GB 42	28	8.5	24.5	32	5.5	1.5
GB 53	30.4	3.6	24	36	5	1
GC 1.6	29	8	23	30	8	2
GC 5.5	22	6	40	27	4.5	0.5
GC 7.4	30	3.2	23	39	4	0.8

The amounts of fatty acids are given as relative percentages of the total peak area

* Cluster 1, fatty acid profile characterized by a similar ratio of C_14:0_ 3-OH and C_28:0_ 27-OH as in the LPS of strain 3841; no label, cluster 2

**Fig. 4:**
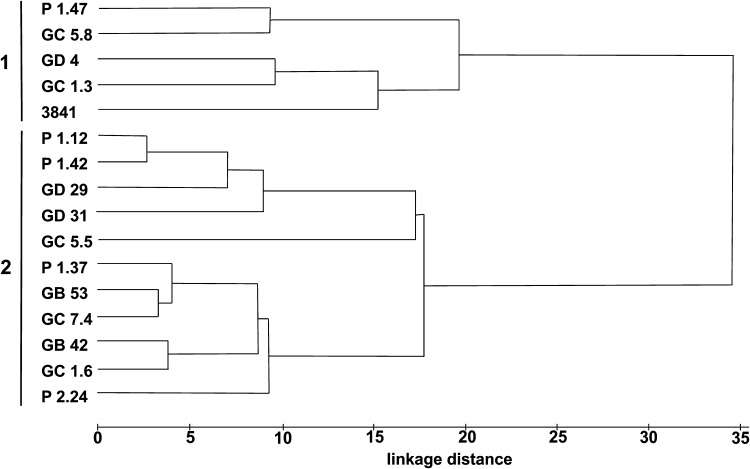
Euclidean distance-based dendrogram of fatty acid (% total) composition derived from the LPS patterns of root pea nodule isolates

A dendrogram based on the mean fatty acid content (% of total fatty acids) confirmed the grouping of fatty acid profiles into two major clusters (Fig. 4; Table 2). In the first cluster, designated as 1 and comprising strains P 1.47, GC 5.8, GD 4, and, GC 1.3, the ratio of fatty acids was similar to that of *R. leguminosarum* 3841; in cluster 2, comprised by the remaining 11 strains, the content of C_15:0_ 3-OH and C_16:0_ 3-OH and *ω*-1 fatty acids was variable (Supplemental Fig. 4).

In addition, variable amounts of nonhydroxy fatty acids, such as hexadecanoic (C_16:0_), octadecanoic (C_18:0_), and octadecenoic (C_18:1_) fatty acids, were detected in all strains. Low levels of palmitic and stearic acids have been also found in lipid A preparations of *R. leguminosarum* and *R. etli* (Muszyński et al. 2011).

### EPS production

All isolates except strain GC 5.5 produced large amounts of EPS, ranging from 216.7 ± 15.1 µg Glc mg^−1^ protein (isolate GC 1.6) to 453 ± 40.2 µg Glc mg^−1^ protein (isolate P 1.42), with an average of 280 µg Glc mg^−1^ protein. Isolate GC 5.5, which exhibited a moderately mucoid colony morphology, produced 2.5 times less EPS (113.7 ± 16.3 µg Glc mg^−1^ protein) than average (Table 1).

### Sensitivity to salt, detergents, pH, and elevated temperature

Features such as sensitivity to NaCl and detergents are usually analyzed to determine whether some environmental conditions might impact the natural selection of strains nodulating legume plants (Zahran 1999; Bolanos et al. 2003).

The analysis of NaCl tolerance revealed moderate resistance to salinity (0.01–1.0% NaCl) of most strains. Four strains, GD 29, GB 53, GC 1.3, and GC 1.6, with group A LPS pattern, were more tolerant and grew at 3.0% NaCl (Table 1).

Half of the evaluated pea microsymbionts, including strain 3841, grew over a relatively wide range of pH values (pH 4.5–7.5), with an optimum at pH 7.2. The remaining isolates grew well at higher pH values, i.e., pH 4.5–8.0 **(**Table 1). Strain 5.5 that produced the lowest amount of EPS grew at a narrow pH range, pH 7.2–8.0.

All the isolates grew at temperatures from 20 to 28 °C, with an optimum at 28 °C. Although all strains survived at 37 and 42 °C, no strain grew at these two temperatures (data not shown).

Most strains isolated from pea nodules were more sensitive to SDS than to DOC. The concentration of SDS that inhibited the growth of the isolates varied from 0.015 to 0.12% (w/v), whereas the inhibitory concentrations of DOC were two times higher, i.e., 0.015–0.25% (Table 1). Strains P 2.24, P 1.12, and 3841 were able to grow only at the lowest tested concentration of SDS and DOC (0.06%).

A correlation between the ability to grow in the presence of higher salt concentrations and the LPS type was apparent in strains with type A LPS. Strains with type A LPS could all grow at similar concentrations of DOC and SDS; detergent sensitivity of strains with type B LPS was more variable. On the other hand, high sensitivity to detergents observed for strains 3841, P 2.24, and GC 5.5 (which all produce lower amounts of EPS) was in accordance with earlier data that indicated that EPS biosynthesis influences strain sensitivity to surface-active detergents (Janczarek et al. 2010). Similarly, according to Vanderlinde et al. (2010), the differences in strain sensitivity to anionic detergents can be linked to the composition of fatty acyl chains in LPS.

**Fig. 5:**
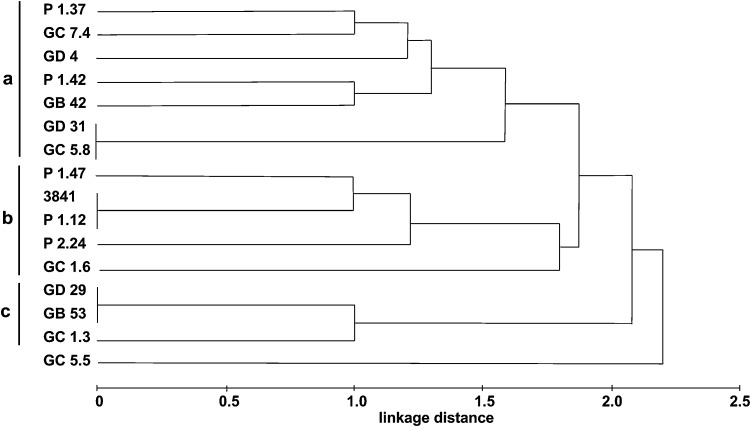
Euclidean distance-based dendrogram presenting phenetic relationships (Table 1) between pea nodule isolates

A dendrogram showing phenetic relationships between the studied strains, based on their sensitivity to salt, SDS, DOC, and pH, is presented in Fig. 5. At least three main strain groups can be distinguished. Group a includes seven isolates, and groups b and c comprise five and three strains, respectively (Fig. 5). Isolate GC 5.5 was unrelated to other strains, probably because of weak pH tolerance. Interestingly, five out of seven strains from phenetic group a also belonged to AFLP I clade, whereas the isolates from groups b and c were dispersed between AFLP groups II and III or remained ungrouped after AFLP profiling (Figs. 1, 5).

## Discussion

A previous investigation of a local population of pea microsymbionts based on PCR–RFLP analysis of 16–23S rRNA ITS and *nodA*–*F* regions, plasmid patterns, and metabolic capabilities showed that individual plants were infected by numerous and diverse strains (Wielbo et al. 2011). The isolates from pea nodules were most closely related to *R. leguminosarum* bv*. viciae* 3841 and *Rhizobium fabae* CCBAU33202 (Wielbo et al. 2011), whereas *P. sativum* isolate GB 30 was most closely related to *R. leguminosarum* bv. *trifolii* WSM1689 (Mazur et al. 2015). In this paper, the strains nodulating *P. sativum* showed greatest similarity to *R. leguminosarum* biovars *viciae* and *trifolii.* Genotypic characteristics of the strains were compared with their electrophoretic and fatty acid LPS profiles. The effect of environmental stress conditions, such as salt, detergents, pH, and elevated temperature, on strain growth was quantitatively analyzed and compared with strain classifications based on the genetic and LPS profiles.

The diversity of LPS is generally associated with the variation in the O-antigen gene cluster. It has been well documented that horizontally acquired genes play a key role in defining LPS structure (Lerouge and Vanderleyden 2001; Muszyński et al. 2011). The differences in the electrophoretic patterns of LPS from strains analyzed herein indicated that the strains occupying the nodules of the pea cultivar significantly differ from one another. No similarity was observed between the dendrogram constructed based on the LPS electrophoretic pattern and the phylogenetic tree based on AFLP analysis. This finding is in agreement with the study of Santamaria et al. (1997, 1998), who demonstrated that LPS profiling ensures good discrimination of strains with a ladder-like pattern of LPS, e.g., *Bradyrhizobium* strains, but it is less useful for *Rhizobium* species since their LPS migrates as less distinctive bands.

The profiles of LPS-derived hydroxy fatty acids were reported to be a valuable chemotaxonomic marker for the systematics of the family *Rhizobiaceae* and distinguishing the genus (Yokota and Sakane 1991; Jarvis et al. 1996; Tighe et al. 2000; Zahran et al. 2003; Vanderlinde et al. 2010). The results of the current analysis of LPS-derived fatty acids suggested that the isolates are highly similar to *R. leguminosarum* and *R. etli*. The cladogram constructed based on the weight percentage of total fatty acids (UPGMA) of the isolates did not reflect the similarities between their LPS electrophoretic profiles.

It was reported that the differences in LPS fatty acid composition between the biovars *viciae*, *phaseoli*, and *trifolii* of *R. leguminosarum* were small (Jarvis et al. 1996; Dunfield et al. 1999). Although the variation in fatty acid profiles within a particular biovar of *R. leguminosarum* is not pronounced, it might nonetheless be used to establish the identity of pea nodule occupants (Dunfield et al. 1999). Our results revealed an agreement between fatty acid composition and phylogenetic classification at the genus level. A previous polyphasic taxonomic study of rhizobia from wild legumes (20 strains) demonstrated both an agreement and lack thereof between data clustered based on phenotypic analysis and total cellular fatty acid profiles (Zahran et al. 2003).

Despite close genetic similarity of strains, their LPS and fatty acid profiles are different. Strain classification based on these differences does not correlate with the genetic relationship, although it allows discrimination between the strains. The results are in agreement with the study of Dunfield et al. (1999). Our observation that there were differences in the proportion of 3-hydroxy to ω-1 hydroxy fatty acids suggests that additional studies are required to determine whether this ratio is dependent on the intrinsic heterogeneity of lipid A, is influenced by external conditions, or a combination of the two possibilities.

Based on a previous study (Wielbo et al. 2011), we can assume that strains producing higher amounts of EPS (GB 42, GD 31, and P 1.42) are also more efficient symbionts. The differences in LPS and fatty acid pattern and EPS production among pea isolates could be reflected in their sensitivity to environmental factors, e.g., salinity, pH range, or temperature. Besides these compounds, the tolerance of strains to environmental conditions, e.g., to acidic pH, is regulated by the adaptive response (Graham et al. 1994). The physiological properties of the strains were slightly discriminative (salt tolerance and growth pH range) or not discriminative (temperature growth range) in diversification of the studied isolates.

The relationship between phenotypic and genetic correlations in symbiotic bacteria is not entirely clear. For example, no apparent relationship between the genotypic and phenotypic diversity of rhizobial strains was noted by Xavier et al. (1998). Similar results were reported by Elboutahiri et al. (2010) for *Sinorhizobium* strains nodulating *Medicago sativa*. In contrast, a correlation between the phenotypic and genotypic characteristics of rhizobial strains isolated in situ from *Accacia senegal* nodules was found (Fall et al. 2008; Bakhoum et al. 2014).

Together with the results of our earlier study revealing a large diversity of pea microsymbionts (Wielbo et al. 2010), the current analysis suggests that the genome-level relationship between rhizobial strains in a localized area does not reflect their metabolic profiles but also their surface characteristics. We propose that the correlation seen between the largest AFLP group I and the physiological properties of ca. 30% of strains could be associated with isolate adaptation to the local soil conditions. Genomic relationships are indispensable for microsymbiont classification in light of high heterogeneity of surface compounds, LPS and EPS, observed in rhizobia infecting the same host plant. The LPS and fatty acid patterns, although not directly related to the genetic relationship, can nonetheless be used for strain differentiation.

## Conclusion

The aim of this work was to evaluate whether LPS profiles and LPS-derived fatty acid patterns of rhizobia nodulating *P. sativum* are related to strain phylogeny. The phylogenetic tree constructed on the basis of the concatenated genes—16S rRNA, *recA*, and *atpD*—revealed that all strains belong to *R. leguminosarum* biovars *viciae* and *trifolii*. The pea isolates were divided into two major groups by AFLP; however, no evident correlation with LPS profiles and LPS-derived fatty acid composition was found. A relationship between LPS profiling and phenotypic characteristics, including sensitivity to anionic detergents and salinity, was not apparent.

## Electronic supplementary material

Below is the link to the electronic supplementary material.

Supp Fig. 1 Phylogenetic tree generated using the neighbor-joining method, constructed based on partial sequences of the 16S rRNA gene of pea nodule isolates and closely related *Rhizobium* species. The distances were calculated according to Kimura’s two-parameter correction. Bootstrap analysis was based on 1000 resamplings. Bar, nucleotide substitution per site (TIFF 12534 kb)

Supp Fig. 2 Neighbor-joining tree showing the phylogenetic relationship between the test strains and related species based on *atpD* gene sequences. Bootstrap values (expressed as percentages of 1000 replications) are given at the nodes. Bar, nucleotide substitution per site (TIFF 12446 kb)

Supp Fig. 3 Neighbor-joining tree showing the phylogenetic relationship between the test strains and related species based on *recA* gene sequences. Bootstrap values (expressed as percentages of 1000 replications) are given at the nodes. Bar, nucleotide substitution per site (TIFF 11957 kb)

Supp Fig. 4 Gas chromatograms of methyl ester TMS ethers of fatty acids derived from the LPS of pea isolate GC 5.8, representing a fatty acid profile similar to that of the LPS from strain 3841 (A); GC 1.6, representing the second profile, with different C_16:0_ 3-OH and C_18:0_ 3-OH to ω-1 hydroxy fatty acid ratios (B). *, palmitic acid (C_16:0_); **, TMS derivative of sugar constituents of LPS; ○, octadecenoic acid (C_18:1_); ●, C_19:0_ cyclo, derived from membrane phospholipids; ■, artifacts formed during hydrolysis of ω-1 hydroxy fatty acid (TIFF 17589 kb)
