# Supplementary figures and images for: Electrophoretic profiles of lipopolysaccharides from Rhizobium strains nodulating Pisum sativum do not reflect phylogenetic relationships between these strains
Source: Arch Microbiol. 2017 Apr 6;199(7):1011–21. doi: 10.1007/s00203-017-1374-1 (PMC5548859; doi:10.1007/s00203-017-1374-1)

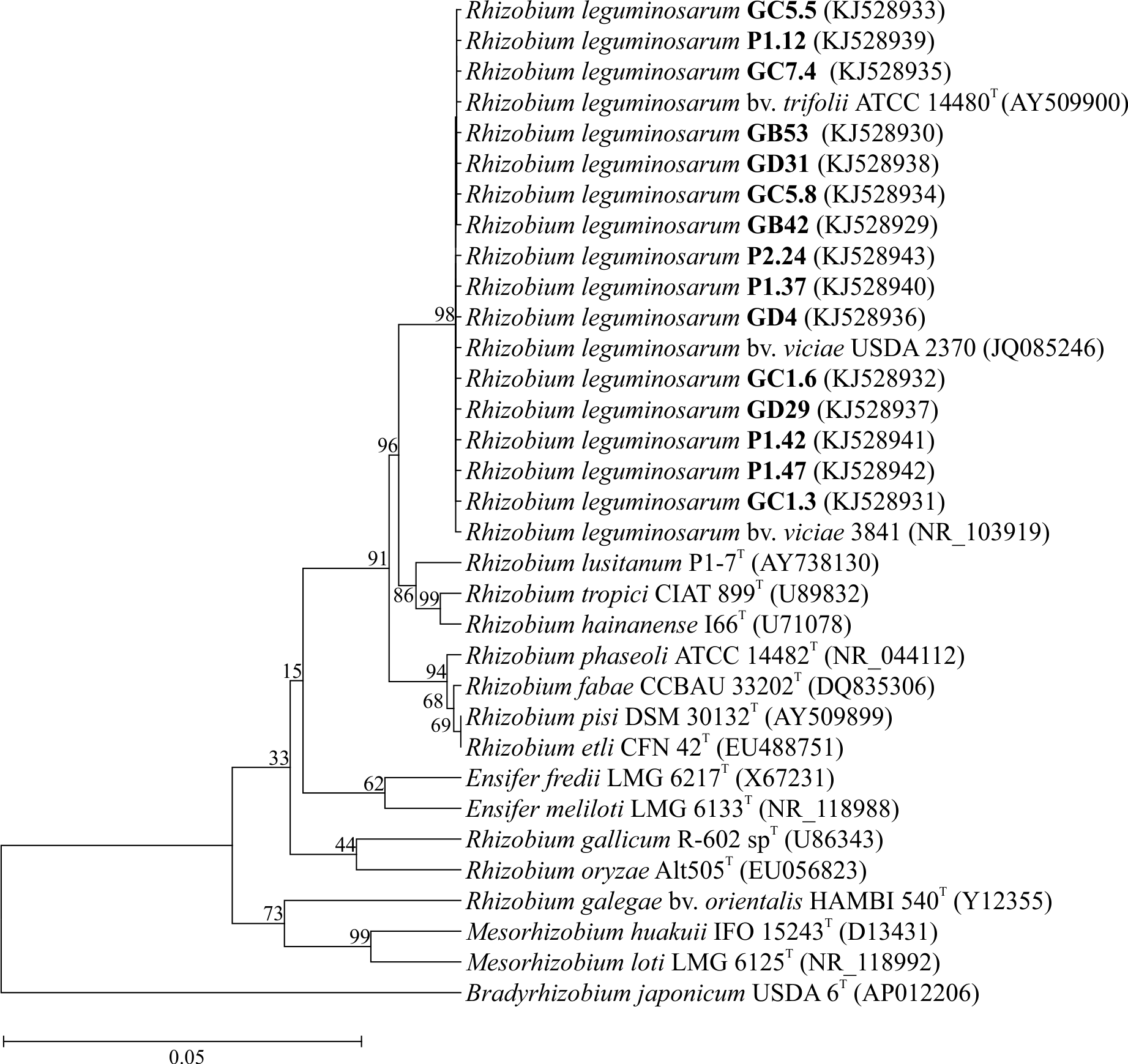

Supplement: Supplementary file 1 — Phylogenetic tree generated using the neighbor-joining method, constructed based on partial sequences of the 16S rRNA gene of pea nodule isolates and closely related Rhizobium species. The distances were calculated according to Kimura’s two-parameter correction. Bootstrap analysis was based on 1000 resamplings. Bar, nucleotide substitution per site (TIFF 12534 kb) [file 203_2017_1374_MOESM1_ESM.tif]

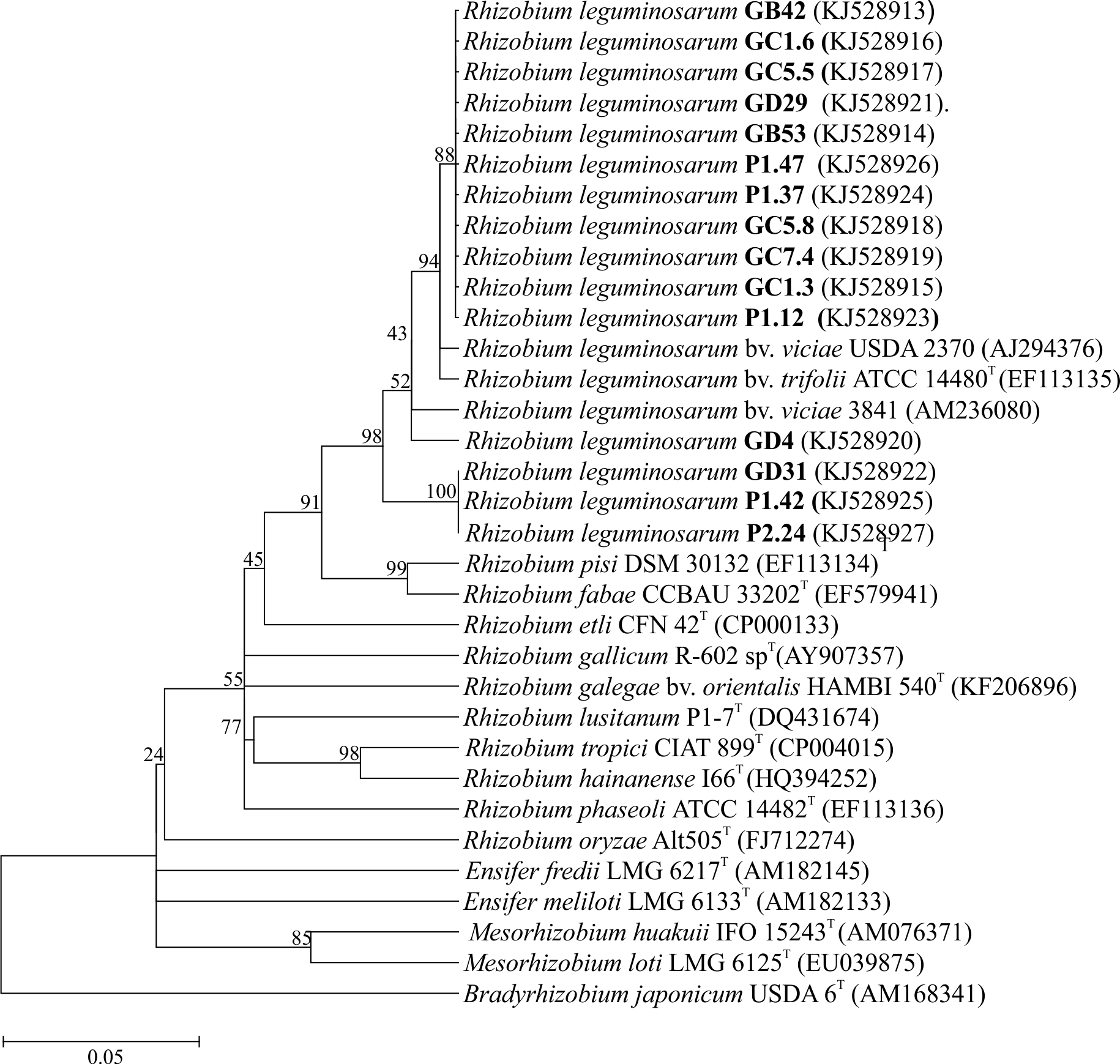

Supplement: Supplementary file 2 — Neighbor-joining tree showing the phylogenetic relationship between the test strains and related species based on atpD gene sequences. Bootstrap values (expressed as percentages of 1000 replications) are given at the nodes. Bar, nucleotide substitution per site (TIFF 12446 kb) [file 203_2017_1374_MOESM2_ESM.tif]

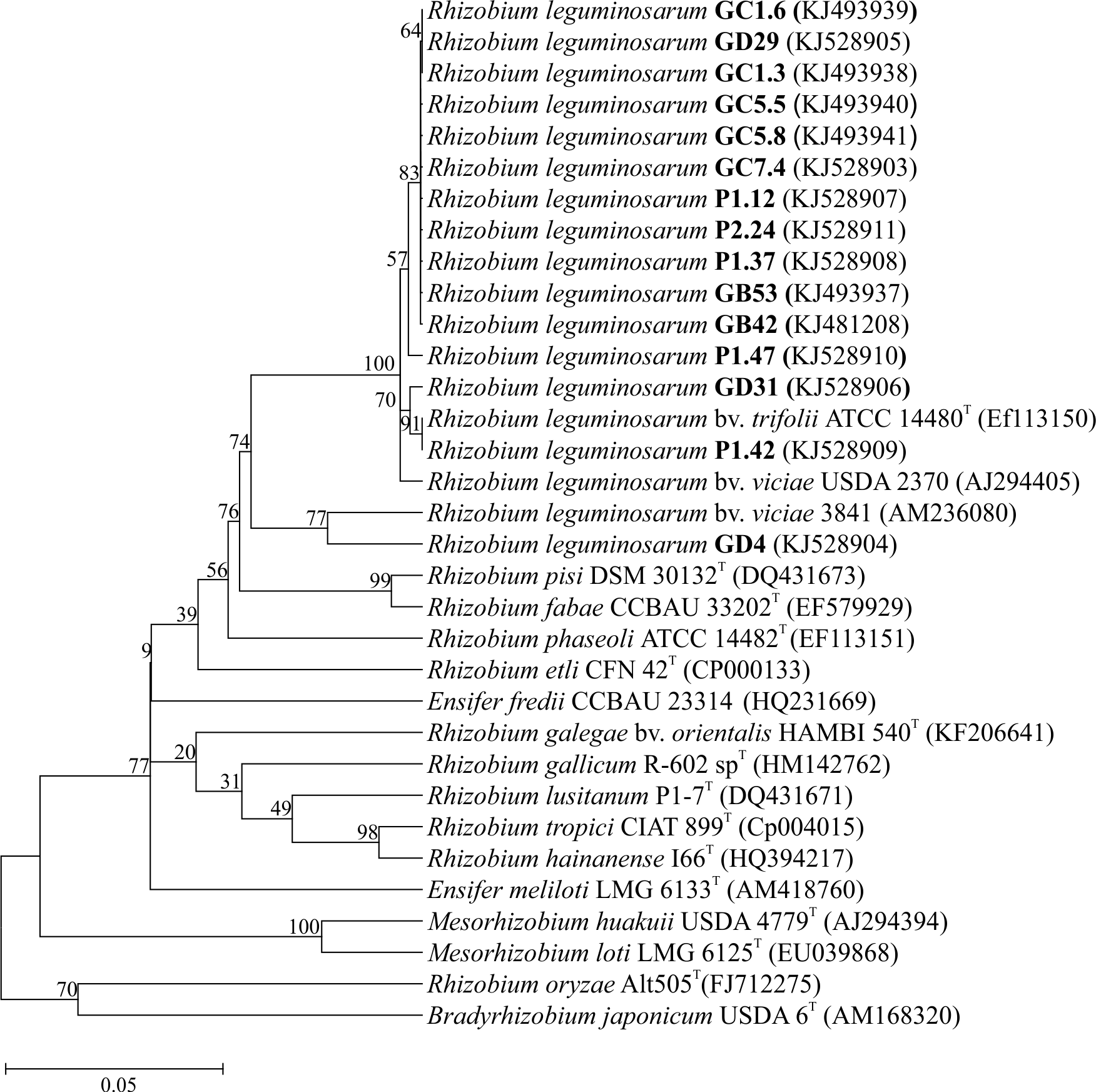

Supplement: Supplementary file 3 — Neighbor-joining tree showing the phylogenetic relationship between the test strains and related species based on recA gene sequences. Bootstrap values (expressed as percentages of 1000 replications) are given at the nodes. Bar, nucleotide substitution per site (TIFF 11957 kb) [file 203_2017_1374_MOESM3_ESM.tif]

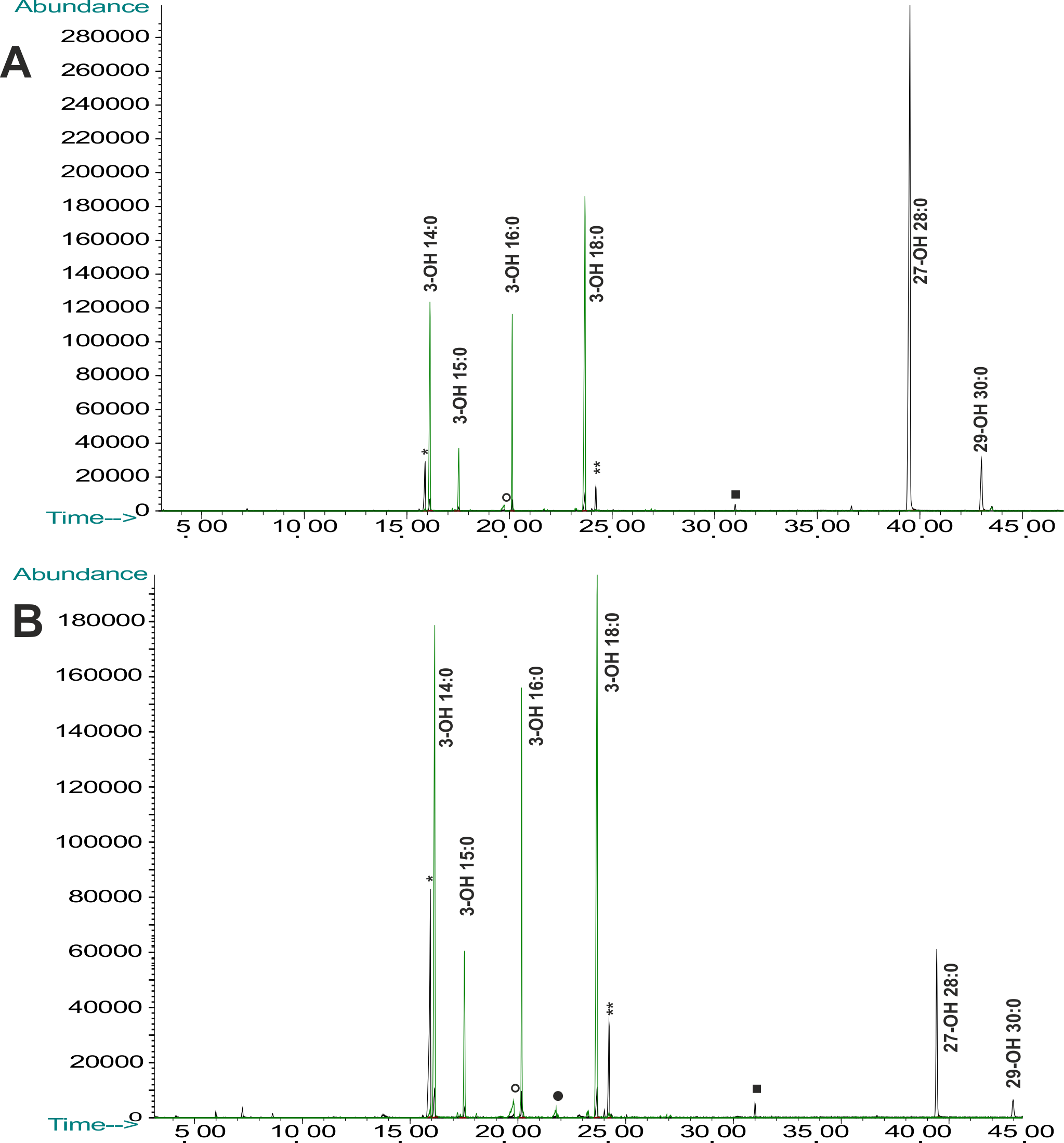

Supplement: Supplementary file 4 — Gas chromatograms of methyl ester TMS ethers of fatty acids derived from the LPS of pea isolate GC 5.8, representing a fatty acid profile similar to that of the LPS from strain 3841 (A); GC 1.6, representing the second profile, with different C16:0 3-OH and C18:0 3-OH to ω-1 hydroxy fatty acid ratios (B). *, palmitic acid (C16:0); **, TMS derivative of sugar constituents of LPS; ○, octadecenoic acid (C18:1); ●, C19:0 cyclo, derived from membrane phospholipids; ■, artifacts formed during hydrolysis of ω-1 hydroxy fatty acid (TIFF 17589 kb) [file 203_2017_1374_MOESM4_ESM.tif]
